# Did smartphones enhance or diminish well-being during the COVID-19 pandemic?

**DOI:** 10.3389/fpsyg.2023.1094196

**Published:** 2023-03-13

**Authors:** Jennifer L. Heyman, Kostadin Kushlev

**Affiliations:** ^1^Department of Psychology, McGill University, Montreal, QC, Canada; ^2^Department of Psychology, Georgetown University, Washington, DC, United States

**Keywords:** smartphones, well-being, COVID-19, pandemic (COVID-19), social distancing

## Abstract

**Introduction:**

As smartphones have become increasingly integrated into people’s lives, researchers have attempted to answer whether they are beneficial or detrimental to well-being. Of particular interest to the current study is the role that smartphones played during the first year of the COVID-19 Pandemic.

**Methods:**

In an intensive longitudinal study, we explore how varying uses of smartphones relate to well-being using the Displacement-Interference-Complementarity framework.

**Results:**

Consistent with pre-pandemic research, we show that people felt better, calmer, and more energetic when they used their phones more for complementary purposes (i.e., to access information, entertainment, and connection not otherwise available). In contrast to most pre-pandemic research, however, we find no evidence that any type of phone use predicted lower well-being during the pandemic.

**Discussion:**

Overall, this study lends support to the idea that smartphones can be beneficial for individuals, particularly during times when face-to-face interaction is limited.

## Introduction

Smartphones have become an essential part of our everyday lives and can play an important role in our well-being. People report using their smartphones for almost every activity including shopping, banking, entertainment, and relationship maintenance (Brown, 2019). Additionally, people report that their smartphones are an integral part of their lives that they could not live without (Perrin, 2017). This reliance on smartphones has become even more apparent during the COVID-19 Pandemic whereby people were required to social distance from one another. However, little research has examined the relationship between smartphone use, social distancing, and well-being (but see Marinucci et al., 2022). Did smartphone use benefit well-being during times of social distancing, acting as a lifeline to connect people and maintain productivity? Or was smartphone use detrimental to well-being during these times by replacing or interfering with other activities?

The current study aims to examine these questions by focusing on how three different forms of smartphone use (i.e., displacement, interference, and complementarity) relate to several indicators of well-being. Furthermore, the study examines the extent of social distancing as a previously unexplored moderator that can shed light on the relationship between smartphone use and well-being.

### Mobile phones and well-being

In recent years, there has been much effort to understand whether and when smartphone use is beneficial or detrimental to our well-being. Much of the previous research has found smartphone use to be related to lower levels of well-being (Khan, 2008; Augner and Hacker, 2012; Hall and Baym, 2012; Gentile et al., 2013; Harwood et al., 2014; Lepp et al., 2015; Roberts and David, 2016). However, the majority of research examining how smartphone use relates to well-being has focused on the amount of time that people spend on their screens: Those who spend more time in front of a screen have been shown to have lower levels of well-being (Przybylski and Weinstein, 2017; Twenge et al., 2018). Importantly, this relationship appears to be nonlinear, with those who spend a moderate amount of time on a mobile device having higher levels of well-being compared to those who spend no time or an excessive amount of time on their devices (Przybylski and Weinstein, 2017; Twenge and Campbell, 2019). Other evidence, however, suggests that the overall effect of screentime on well-being is negligible (Orben and Przybylski, 2019). Thus, recent research has shifted from examining the effects of simple screentime toward examining how the different ways in which people use their smartphones affect well-being.

The Displacement–Interference–Complementarity Framework, for example, posits three distinct mechanisms about how smartphones affect well-being (Kushlev and Leitao, 2020). First, the Displacement Hypothesis posits that phone use will relate to well-being to the extent that it replaces time spent doing other activities. For example, using one’s phone to replace face-to-face interactions—an established factor in higher well-being—may relate to lower levels of well-being (McDaniel et al., 2021). Conversely, phone use should relate to higher levels of well-being if it replaces time spent engaging in activities that are detrimental to well-being, such as ruminating on problems (Li et al., 2021). In this case, the displacement caused by the smartphone would allow for less time spent on the detrimental activity, thus improving one’s well-being.

The second mechanism, termed the Interference Hypothesis, states that phone use will relate to well-being to the extent that it interferes with concurrent activities. Past research suggests that distraction can be an effective emotion regulation strategy at least in the short term, dampening the impact of both positive (Quoidbach et al., 2010) and negative events (Sheppes and Meiran, 2007). Thus, people distracted by their smartphones during face-to-face interactions, for example, have been shown to experience lower levels of well-being (David et al., 2015; Kushlev et al., 2016; Roberts and David, 2016; Dwyer et al., 2018; Kushlev and Dunn, 2019). However, just as with the displacement hypothesis, it is possible that interference from smartphones will benefit well-being if it interferes with harmful activities. For example, if one is ruminating on a problem outside of their control, an alert or notification from their smartphone may break the cycle, thus allowing them to direct their attention to more beneficial activities.

Finally, the Complementarity Hypothesis posits that phone use will relate to well-being to the extent that it provides information or opportunities not otherwise available. For example, using a phone to stay in touch with others who are not geographically close would relate to higher levels of well-being (Neustaedter and Greenberg, 2012; Hampton et al., 2017; Holtzman et al., 2021). It is also possible, though, that complementary phone use would relate to lower levels of well-being if it allows for greater access to information or engagement in activities that are harmful to well-being, such as access to disturbing news stories or receiving negative feedback on a social media post.

Overall, the Displacement–Interference–Complementarity Framework states that smartphones will affect well-being in different ways depending on how and when they are used. The COVID-19 Pandemic drastically changed the ways in which we use our smartphones. That is, our social environments were suddenly changed at the onset of the Pandemic, thereby reducing the activities that phone use could replace or interfere with and enhancing the importance of phone use to maintain social contact. Therefore, it is important to consider how this global phenomenon has influenced the relationship between smartphone use and well-being.

### Mobile phone use during COVID

The onset of the COVID-19 Pandemic drastically altered every aspect of people’s lives around the world. In the United States, a total of 42 states issued an official and mandatory stay-at-home order by the end of May 2020 (Moreland et al., 2020), thereby limiting social interactions with others to (a) those with whom one shares a dwelling or (b) those with whom one interacts *via* digital devices. As a result of this, the importance of mobile phones skyrocketed during this time. Indeed, people appear to be spending more time on their phones compared to pre-pandemic times, with one study finding a 10-h increase in weekly recreational screentime from pre-pandemic to pandemic times in children (McArthur et al., 2021). But how does this increased reliance on smartphones during the COVID-19 Pandemic relate to well-being?

In terms of the Displacement Hypothesis, it is possible that the increased screentime during times of social distancing may relate to lower levels of well-being as research has shown that high levels of screentime predict lower well-being and mental health (Przybylski and Weinstein, 2017; Twenge and Campbell, 2019). Indeed, higher levels of screentime have been shown to be related to poorer mental health during the COVID-19 Pandemic (Smith et al., 2020). Furthermore, smartphone use during the COVID-19 Pandemic has been shown to be related to poorer sleep quality and duration (Islam et al., 2021; Koban et al., 2022), which could have negative effects on well-being (Mac Cárthaigh et al., 2020). However, this effect may not be as prominent during the COVID-19 Pandemic, seeing as those who work from home experience greater flexibility in their schedules and are not bound by the typical 8-h workday (Fukumura et al., 2021; Routley, 2021; Koban et al., 2022). It is also possible that smartphone use may increase levels of well-being as people use it as an escape from the stressful life events surrounding the COVID-19 Pandemic (de Freitas et al., 2022; Potas et al., 2022). That is, people may be using their smartphones to replace time spent ruminating on the personal and global issues surrounding the Pandemic, thus mitigating the effects of COVID-19 related rumination.

Similarly, smartphone interference has been shown to be related to lower levels of well-being (David et al., 2015; Kushlev et al., 2016; Roberts and David, 2016; Kushlev and Dunn, 2019). For example, work-related email notifications have been shown to interfere with leisure activities outside of work hours (Derks and Bakker, 2014; Derks et al., 2015), which can reduce levels of well-being and the quality of time spent with family (Belkin et al., 2016). This effect could be heightened during the COVID-19 Pandemic as the lines between work and leisure have become less distinct (Routley, 2021). However, just as with the Displacement Hypothesis, the increased schedule flexibility, both in work and personal lives, may result in a reduced perception of interference, thus mitigating the negative effects of smartphone interference on well-being.

Finally, in line with the Complementarity Hypothesis, mobile phone use could benefit well-being by giving people the opportunity to interact with others when face-to-face interaction is not available. Indeed, people have been able to receive sufficient social support *via* online interactions during the pandemic, thus increasing levels of well-being (Canale et al., 2020). Furthermore, David and Roberts (2021) found that social distancing was related to lower levels of social connection and well-being, but only for those who had low levels of smartphone use. In contrast, those who used their smartphones more frequently did not experience lower levels of social connection and well-being as a result of social distancing. In other words, smartphone use mitigated the negative effect of social distancing on well-being. Consistent with the Complementarity Hypothesis, then, smartphone use may relate to higher levels of well-being by providing access to social support and communication that would otherwise not be available due to the social distancing measures during the COVID-19 Pandemic. However, it is also possible that the increased access to information related to the COVID-19 Pandemic afforded by smartphones will relate to lower levels of well-being. Indeed, previous research has found that the constant access to news about the COVID-19 Pandemic was related to higher levels of anxiety and psychological distress (Stainback et al., 2020). Therefore, it is possible that using one’s smartphone to access information that would otherwise not be available would relate to lower levels of well-being if the information that is being provided is distressing.

### The present research

While there is a large amount of interest in examining the role of digital media during the COVID-19 Pandemic, no research has examined the moderating role of social distancing in the relationship between smartphone use and well-being. As such, the current study focuses on two primary research questions:

RQ1: How does mobile phone use relate to well-being during the COVID-19 Pandemic?
RQ2: What is the role of social distancing in the relationship between mobile phone use and well-being?

To gain a cohesive understanding of how smartphone use relates to well-being, we examine smartphone use through the lens of the Displacement-Interference-Complementarity Hypothesis. That is, how does the extent to which phone use (a) displaces time spent doing other activities, (b) interferes with concurrent activities, and (c) complements concurrent activities relate to well-being, and what is the role of social distancing in these relationships? Much of the previous research has found displacement and interference to predict lower levels of well-being during pre-pandemic times (David et al., 2015; Kushlev et al., 2016; Roberts and David, 2016; Przybylski and Weinstein, 2017; Kushlev and Dunn, 2019; Twenge and Campbell, 2019), but will these negative effects still be present during the COVID-19 Pandemic? In contrast, much of the previous research has found phone complementarity to predict higher levels of well-being (Neustaedter and Greenberg, 2012; Hampton et al., 2017; Holtzman et al., 2021). Will this effect remain positive during the COVID-19 Pandemic, given the importance of smartphones to maintain contact with others? Or will it be more negative given the constant access to distressing information that is afforded by smartphones?

To answer these questions, we conducted a longitudinal study examining the relationships between smartphone use, well-being, and social distancing during the COVID-19 Pandemic. Using a community-based sample, we conducted weekly surveys over a six-month period to examine the extent to which smartphone use relates to well-being and the role that social distancing plays in this relationship. This study was pre-registered^1^ and all data and exclusions can be found here.^2^ Based on our preregistered power analyses, we aimed to recruit at least 200 participants reporting at least 779 episodes overall.

## Methods

### Procedure

Participants were asked to complete a baseline survey assessing demographics, mobile phone use habits, social distancing measures, and well-being. The baseline survey also included our primary measures of interest in this report, including mobile phone use and social distancing over the previous 24 h. Participants then completed brief surveys every 2 weeks for a total of 11 surveys assessing their well-being, mobile phone use, and social distancing over the previous 24 h. The final survey was administered in October 2020^3^.

### Participants

A total of 202 people were recruited through Mechanical Turk in May 2020 for this study (112 man/trans-man, 82 woman/trans-woman, 8 other; *M*_age_ = 37.37, SD_age_ = 11.10; for detailed description of *a priori* power analysis).^4^ In total, 132 participants completed at least two of the brief weekly surveys (70 men/trans-men, 58 women/trans-women, 4 other; *M*_age_ = 40.02, *SD*_age_ = 12.64); 41 participants completed the final, 11th weekly survey (19 men/trans-men, 21 women/trans-women, 1 other; *M*_age_ = 40.49, SD_age_ = 13.38); 11 participants completed all 12 surveys (4 men/trans-men, 6 women/trans-women, 1 other; *M*_age_ = 49.82, SD_age_ = 13.88). To see a more detailed description of participant demographics for each week, see Tables 1A and 1B.

**Table 1A:** Demographic data for participants who completed each week of surveys.

Survey number	*N*	*M*_age_ (SD_age_)	Gender
Baseline	202	37.37 (11.10)	112 man/trans-man, 82 woman/trans-woman, 8 other
Week 2	75	39.51 (11.71)	36 man/trans-man, 35 woman/trans-woman, 4 other
Week 3	59	39.15 (12.99)	24 man/trans-man, 32 woman/trans-woman, 3 other
Week 4	79	39.71 (12.69)	35 man/trans-man, 40 woman/trans-woman, 4 other
Week 5	66	39.71 (12.69)	33 man/trans-man, 31 woman/trans-woman, 2 other
Week 6	59	40.66 (12.67)	29 man/trans-man, 27 woman/trans-woman, 3 other
Week 7	44	41.95 (12.48)	21 man/trans-man, 22 woman/trans-woman, 1 other
Week 8	42	42.05 (12.92)	19 man/trans-man, 22 woman/trans-woman, 1 other
Week 9	34	43.35 (13.14)	15 man/trans-man, 18 woman/trans-woman, 1 other
Week 10	40	41.48 (13.63)	19 man/trans-man, 20 woman/trans-woman, 1 other
Week 11	41	40.73 (13.64)	20 man/trans-man, 20 woman/trans-woman, 1 other
Week 12	41	40.49 (13.38)	19 man/trans-man, 21 woman/trans-woman, 1 other

**Table 1B:** Demographic data for participants who completed each number of surveys.

Survey count	*N*	*M*_age_ (SD_age_)	Gender
1	202	37.37 (11.10)	112 man/trans-man, 82 woman/trans-woman, 8 other
2	132	37.75 (11.70)	70 man/trans-man, 58 woman/trans-woman, 4 other
3	103	38.29 (11.76)	54 man/trans-man, 45 woman/trans-woman, 4 other
4	79	39.77 (12.23)	41 man/trans-man, 34 woman/trans-woman, 4 other
5	59	39.75 (12.63)	27 man/trans-man, 30 woman/trans-woman, 2 other
6	57	41.06 (12.86)	22 man/trans-man, 27 woman/trans-woman, 2 other
7	41	42.68 (13.14)	17 man/trans-man, 23 woman/trans-woman, 1 other
8	35	43.91 (13.40)	13 man/trans-man, 21 woman/trans-woman, 1 other
9	28	46.50 (13.18)	9 man/trans-man, 18 woman/trans-woman, 1 other
10	22	45.59 (14.47)	7 man/trans-man, 14 woman/trans-woman, 1 other
11	19	45.42 (15.21)	6 man/trans-man, 12 woman/trans-woman, 1 other
12	11	49.82 (14.50)	4 man/trans-man, 6 woman/trans-woman, 1 other

## Measures

### Phone Use

#### Displacement

Our operationalization was based on the premise that time is a finite resource, so any time people spend on their phones is time they do not spend doing something else. Phone displacement was measured with three items. First, screentime in bed was measured with the item “How much time did you spend on a screen IN BED before falling asleep?” (in Hours: *M* = 0.80, SD = 0.55). Phone overuse was measured with the item “In the past 24 h, I spent more time on my phone than I wanted to” using a 1 (not at all) to 7 (very much) scale (*M* = 3.60, SD = 1.76). Finally, total screentime was measured with the item “In the past 24 h, how much time did you spend in front of a screen across all your devices, NOT including time for work or homework (in hours)?” (*M* = 5.66, SD = 3.13). Although this item does not examine phone use directly, previous research examining the displacement hypothesis has examined overall screentime (Hiltunen et al., 2021). These items were weakly to moderately correlated with one another (0.32 < *r*s < 0.57) and internal consistency was good (*α* = 0.69). Therefore, these items were combined to form a single standardized “displacement” item.

#### Interference

Phone interference was measured with three items. Exogenous distraction was measured with the item “In the past 24 h, how often did you get distracted by alerts and notifications?” using a 1 (never) to 5 (very often) scale (*M* = 2.62, SD = 1.12). Endogenous distraction was measured with the item “In the past 24 h how often did you get distracted by checking your phone (without being prompted by a notification)?” using the same 1 (never) to 5 (very often) scale (*M* = 2.64, SD = 1.08). Finally, total phone distraction was measured with the item “In the past 24 h, how often did your phone fragment your attention on other tasks and activities?,” again using the same 1 (never) to 5 (very often) scale (*M* = 2.69, SD = 1.15). These items were all highly correlated with one another (0.62 < *r*s < 0.68) and showed high internal consistency (*α* = 0.84), so they were combined to form a single standardized “interference” item.

#### Complementarity

Phone complementarity was assessed with three items. Phone information was measured with the item “In the past 24 h, my phone allowed me to access information when I needed it (e.g., news, weather, direction, reviews, etc.)” using a 1 (not at all) to 5 (very much) scale (*M* = 3.63, SD = 1.03). Phone entertainment was measured with the item “In the past 24 h, my phone provided a source of entertainment (e.g., videos, games, etc.)” using the same 1 (not at all) to 5 (very much) scale (*M* = 3.30, SD = 1.14). Finally, phone communication was measured with the item “In the past 24 h, my phone allowed me to talk with people I would otherwise be unable to reach (e.g., friends and family who live far away),” again using the same 1 (not at all) to 5 (very much) scale (*M* = 3.53, SD = 1.11). These items were all moderately correlated with one another (0.41 < *r*s < 0.50) and showed high internal consistency (*α* = 0.72), so they were combined to form a single standardized “complementarity” item.

### Well-being

Participants completed three items assessing their well-being (Schimmack and Grob, 2000). Specifically, this scale assesses a three-dimensional model of affect. First, participants were asked to indicate how they were feeling over the past 24 h using a-3 (very bad) to 3 (very good) scale (*M* = 1.05, SD = 1.42). This item has been used in previous research to examine current mood (e.g., Killingsworth and Gilbert, 2010). We refer to this item as ‘feeling good’ and used it as our primary indicator of well-being.

In addition to current mood, as per Schimmack and Grob (2000), we measured two other aspects of affect. Tense arousal was measured by asking participants to indicate how they were feeling over the past 24 h using a − 3 (very tense/anxious) to 3 (very relaxed/calm) scale (*M* = 0.65, SD = 1.49). Similarly, energetic arousal was measured by asking participants to indicate how they were feeling using a—3 (very tired) to 3 (full of energy) scale (*M* = 0.67, SD = 1.59). We refer to these items as “feeling calm,” and “feeling energetic,” respectively.

### Social distancing

Participants were asked to indicate the extent to which they practiced social distancing over the past 24 h using a 1 (not at all) to 4 (completely) scale (*M* = 3.12, SD = 0.89).

## Data analytic procedure

As per the preregistration, we examined both within-and between-person effects of the extent to which phone displacement, interference, and complementarity relate to well-being. To calculate between-person variables, we first calculated each person’s average level of displacement, interference, and complementarity across the six-month study period (their person-mean) before grand-mean centering these scores. The between-person variables thus indicate the extent to which each person’s phone displacement, interference, and complementarity compare to the average across the entire sample. Within-person variables were calculated by subtracting each participant’s person-mean from their raw level of displacement, interference, and complementarity each week. The within-person variables thus indicate the extent to which each person’s phone displacement, interference, and complementarity compare to their unique average across the six-month study period.

To examine how phone use relates to well-being, we used a multilevel model using R’s (R Development Core Team, 2015) lme4 package (Bates et al., 2014) using the following equation:

Equation 1a:

$$\begin{array}{l} {\mathrm{WellBeing}}_{p}={ß}_{\mathrm{0p}}+{ß}_{\mathrm{1p}}{\mathrm{DisplacementWithin}}_{p}\\ +{ß}_{\mathrm{2p}}{\mathrm{DisplacementBetween}}_{p}\\ +{ß}_{\mathrm{3p}}{\mathrm{InterferenceWithin}}_{p}\\ +{ß}_{\mathrm{4p}}{\mathrm{InterferenceBetween}}_{p}\\ +{ß}_{\mathrm{5p}}{\mathrm{ComplementarityWithin}}_{p}\\ +{ß}_{\mathrm{6p}}{\mathrm{ComplementarityBetween}}_{p}\\ +{ß}_{\mathrm{7p}}{\mathrm{Time}}_{p}+e_{p}\;\\ {ß}_{\mathrm{0p}}=\gamma_{00}+U_{\mathrm{0p}}. \end{array}$$

Here, WellBeing*_p_* represents participant *p*’s well-being (as indicated by the extent to which they felt either good, calm, or energetic). ß_1p_DisplacementWithin_p_, ß_3p_InterferenceWithin_p_, and ß_5p_ComplementarityWithin_p_ represent the within-person effects of phone displacement, interference, and complementarity on well-being. That is, does participant *p*’s well-being differ on days when their phone displacement, interference, or complementarity is different than their own average level across the experimental period? Similarly, ß_2p_DisplacementBetween_p_, ß_4p_InterferenceBetween_p_, and ß_6p_ComplementarityBetween_p_ represent the between-person effects of phone displacement, interference, and complementarity on well-being. That is, does participant *p*’s well-being differ for those who have higher levels of phone displacement, interference, or complementarity compared to others in the study? Finally, ß_7p_Time_p_ represents the change in well-being over the course of the experimental period. We allowed intercepts to vary by participant. However, due to convergence issues, we did not allow slopes to vary randomly by participant. To examine the role of social distancing in the relationship between phone use and well-being, we added within-and between-centered social distancing as a moderator of each slope in the models using the following equation:

Equation 1b:

$$\begin{array}{c} {\mathrm{WellBeing}}_{p}={ß}_{\mathrm{0p}}+{ß}_{\mathrm{1p}}{\mathrm{DisplacementWithin}}_{p}\\ +{ß}_{\mathrm{2p}}{\mathrm{DisplacementBetween}}_{p}\\ +{ß}_{\mathrm{3p}}{\mathrm{InterferenceWithin}}_{p}\\ +{ß}_{\mathrm{4p}}{\mathrm{InterferenceBetween}}_{p}\\ +{ß}_{\mathrm{5p}}{\mathrm{ComplementarityWithin}}_{p}\\ +{ß}_{\mathrm{6p}}{\mathrm{ComplementarityBetween}}_{p}\\ +{ß}_{\mathrm{7p}}{\mathrm{Time}}_{p}+e_{p} \end{array}$$

$${ß}_{\mathrm{0p}}=\gamma_{00}+U_{\mathrm{0p}}$$

$$\beta_{\mathrm{1p}}=\gamma_{10}+\gamma_{11}{\mathrm{SocialDistanceWithin}}_{p}+U_{\mathrm{1p}}$$

$$\beta_{\mathrm{2p}}=\gamma_{20}+\gamma_{21}{\mathrm{SocialDistanceBetween}}_{p}+U_{\mathrm{2p}}$$

$$\beta_{\mathrm{3p}}=\gamma_{30}+\gamma_{31}{\mathrm{SocialDistanceWithin}}_{p}+U_{\mathrm{3p}}$$

$$\beta_{\mathrm{4p}}=\gamma_{40}+\gamma_{41}{\mathrm{SocialDistanceBetween}}_{p}+U_{\mathrm{4p}}$$

$$\beta_{\mathrm{5p}}=\gamma_{50}+\gamma_{51}{\mathrm{SocialDistanceWithin}}_{p}+U_{\mathrm{5p}}$$

$$\beta_{\mathrm{6p}}=\gamma_{60}+\gamma_{61}{\mathrm{SocialDistanceBetween}}_{p}+U_{\mathrm{6p}}$$

Here, social distancing was added as a moderator of the displacement (γ_11_, γ_21_), interference (γ_31_, γ_41_), and complementarity (γ_51_, γ_61_) slopes. Positive interactions would therefore indicate that higher levels of phone displacement, interference, or complementarity was related to higher levels of well-being during times of higher social distancing. Negative interactions would indicate that higher levels of phone displacement, interference, or complementarity was related to higher levels of well-being during times of lower social distancing.

In addition to estimating the associations of each predictor with well-being while controlling for the other two predictors in MLM models, we also estimated the bivariate correlations between each predictor and outcome. The within-subjects correlations are available in Table S1a and the between-subjects correlations are available in Table S1b (see Supplementary Online Materials). Finally, because we had three outcome measures—feeling good, calm, and energetic—we used Bonferroni corrections for all our *p*-values, whereby we multiplied *p* by 3 to reduce the incidence of Type 1 error.

## Results

### How does mobile phone use relate to well-being during the COVID-19 pandemic?

**Figure 1:**
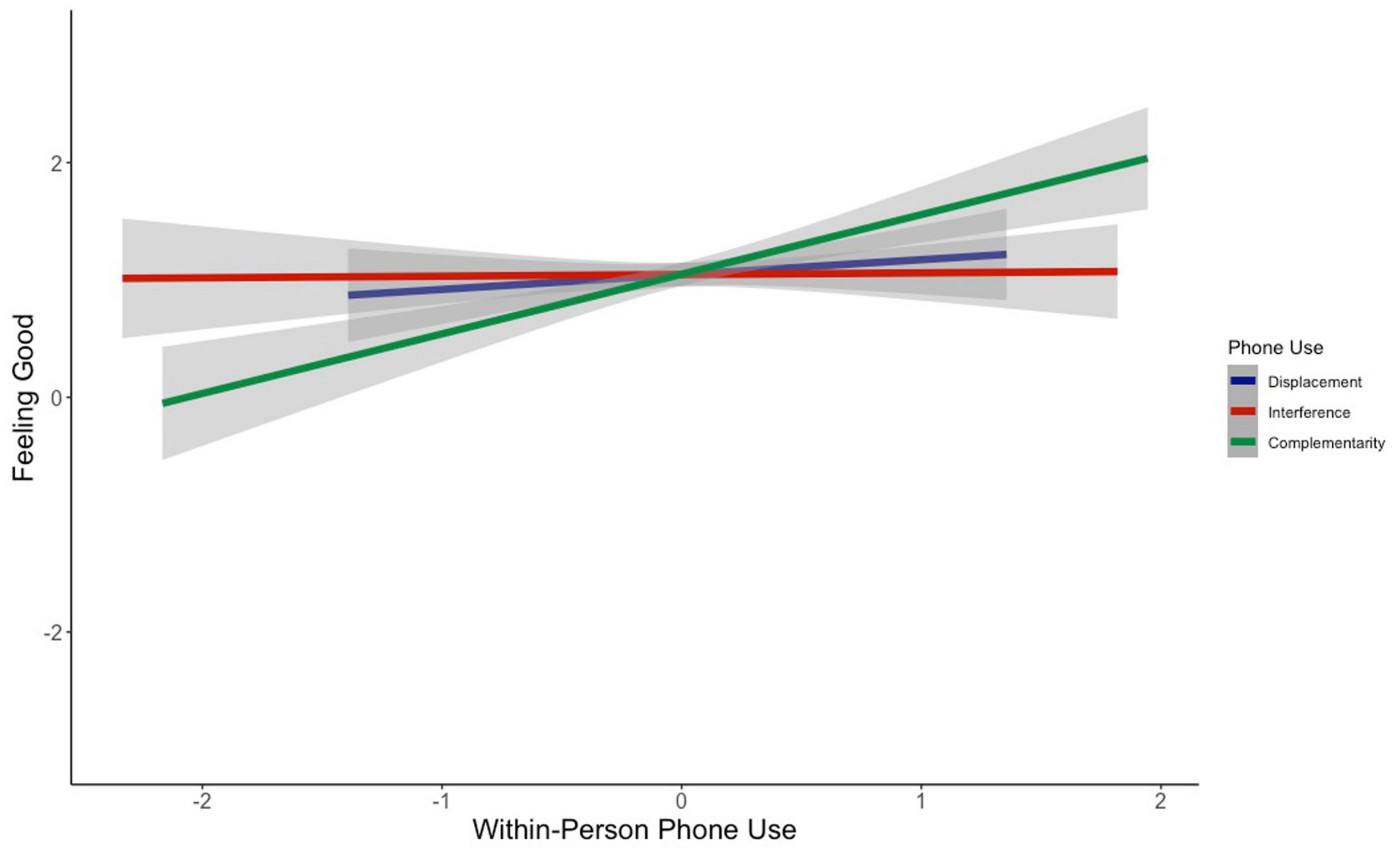
Association between within-person phone use and feeling good.

**Figure 2:**
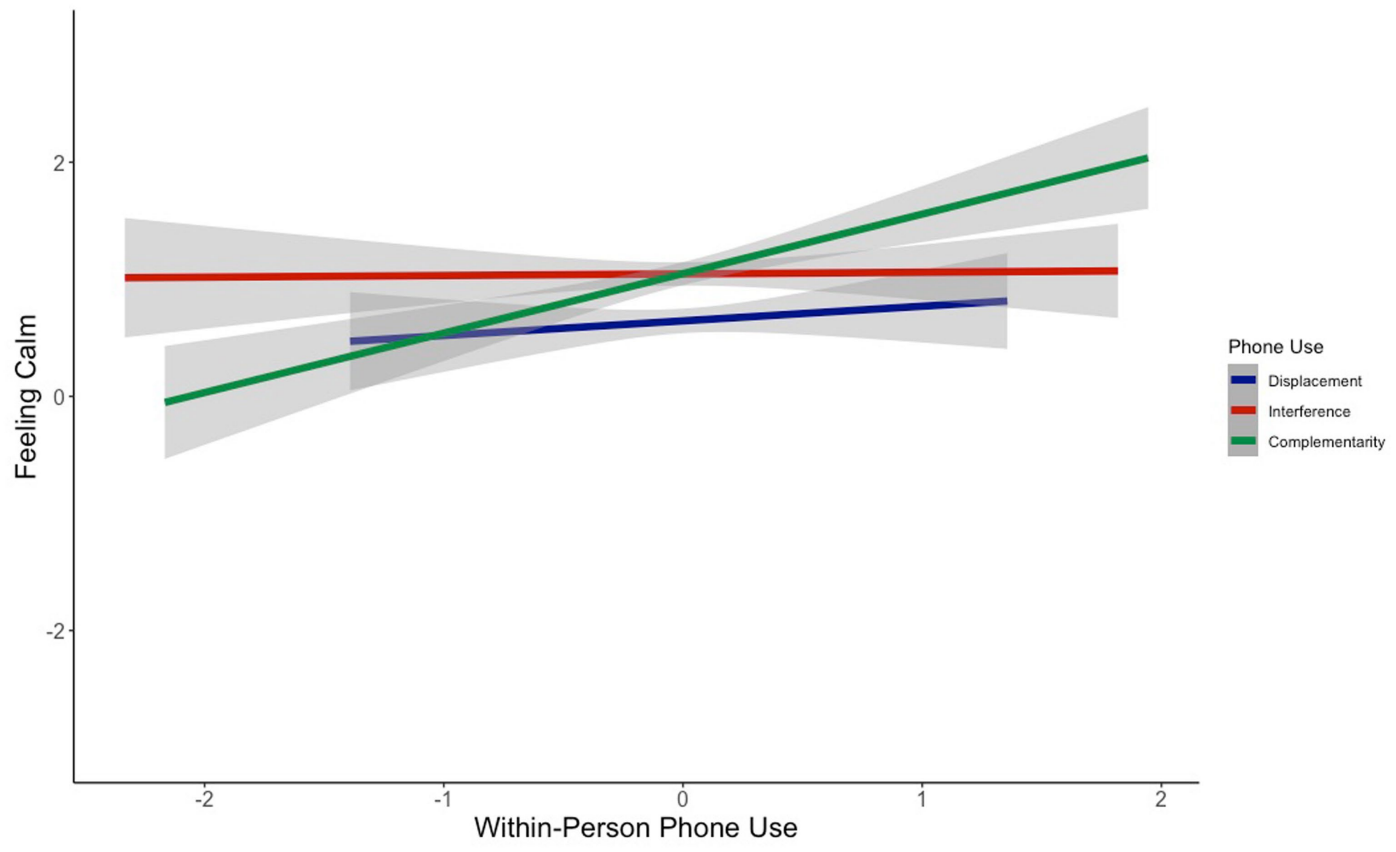
Association between within-person phone use and feeling calm.

**Figure 3:**
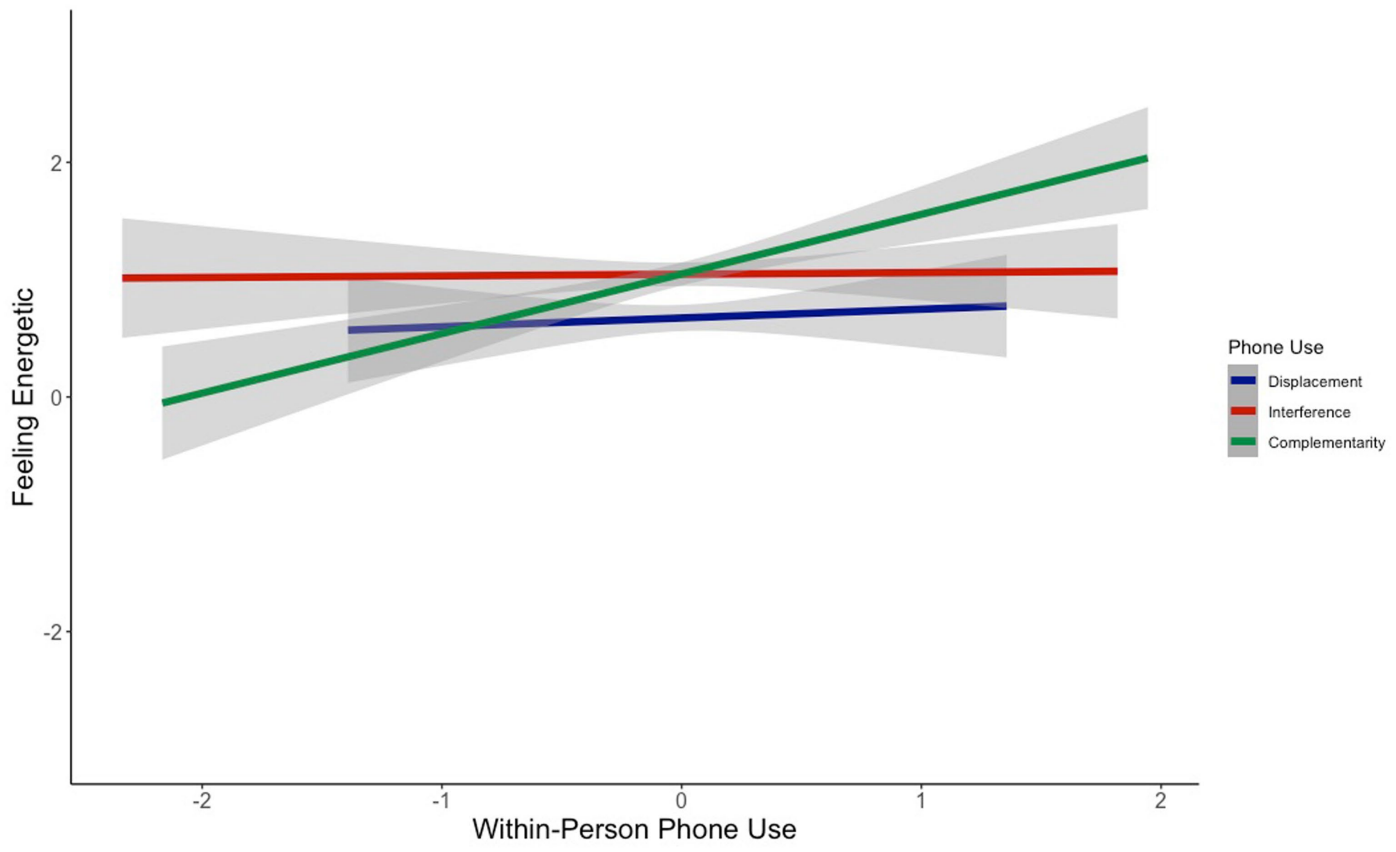
Association between within-person phone use and feeling energetic.

Does the way in which people use their phones influence how they feel? At the within-person level, we found that phone complementarity was significantly positively associated with feeling good (*b* = 0.67, *z* = 5.88, *p*_bonf_ < 0.0001; Figure 1), feeling calm (*b* = 0.38, *z* = 3.85, *p*_bonf_ = 0.0004; Figure 2), and feeling energetic (*b* = 0.44, *z* = 4.79, *p*_bonf_ < 0.0001; Figure 3), while controlling for displacement and interference (see Table 2). That is, on days when people reported using their phones for more complementary purposes, they reported better mood, feeling calmer, and feeling more energetic. These effects were small-to-medium in size (Table 2). In contrast, indicators of phone displacement and interference were not significantly associated with feeling good (Figure 1), feeling calm (Figure 2), or feeling energetic (Figure 3; Table 2).

**Table 2:** Within-person associations between phone use indicators and feeling good, calm, and energetic.

	Feeling good (*n*_episode_ = 782)			Feeling calm (*n*_episode_ = 781)			Feeling energetic (*n*_episode_ = 782)		
	*b* (*se*)	*z*	*r* [95%CI]	*b* (*se*)	*z*	*r* [95%CI]	*b* (*se*)	*z*	*r* [95%CI]
Displacement	0.13 (0.109)	1.20	0.01 [−0.08, 0.34]	0.14 (0.113)	1.24	0.04 [−0.08, 0.36]	0.04 (0.104)	2.45	0.01 [−0.16, 0.25]
Interference	−0.11 (0.095)	−1.14	0.06 [−0.29, 0.08]	−0.11 (0.100)	−1.17	0.04 [−0.31, 0.08]	0.14 (0.091)	1.59	0.06 [−0.03, 0.32]
Complementarity	0.56^***^ (0.100)	5.88	0.04 [0.38, 0.75]	0.38^**^ (0.100)	3.85	0.14 [0.19, 0.57]	0.44^***^ (0.092)	4.79	0.17 [0.26, 0.62]

*b* = unstandardized regression coefficient; SE = standard error. *r* = Pearson’s Correlation Coefficient. 95% confidence intervals for bs are provided. ^***^p_bonf_ < 0.001, ^**^p_bonf_ < 0.01.

We found a similar pattern at the between-person level, such that phone complementarity was significantly positively associated with feeling good (*b* = 0.71, *z* = 6.17, *p_bonf_* < 0.0001), feeling calm (*b* = 0.71, *z* = 5.93, *p*_bonf_ < 0.0001), and feeling energetic (*b* = 0.52, *z* = 3.83, *p_bonf_* = 0.0005), while controlling for displacement and interference. In other words, those who reported using their phones for more complementary purposes compared to others in the study reported better mood, feeling calmer, and feeling more energetic. These effects were medium-to-large (Table 3). Similarly, phone displacement was significantly positively associated with feeling energetic at the between-person level (*b* = 0.42, *z* = 2.45, *p*_bonf_ = 0.05), although it was not significantly associated with feeling good (*b* = 0.20, *z* = 1.38, *p*_bonf_ = 0.51) or feeing calm (*b* = 0.19, *z* = 1.25, *p* = 0.64; Table 2). Phone interference was not significantly associated with feeling good (*b* = −0.14, *z* = −1.02, *p_bonf_* = 0.93), feeling calm (*b* = −0.15, *z* = −1.05, *p*_bonf_ = 0.88), or feeling energetic (*b* = −0.08, *z* = −0.49, *p*_bonf_ > 0.99).

**Table 3:** Between-person associations between phone use indicators and feeling good, calm, and energetic.

	Feeling good			Feeling calm			Feeling energetic		
	*b* (se)	*z*	*r* [95%CI]	*b* (se)	*z*	*r* [95%CI]	*b* (se)	*z*	*r* [95%CI]
Displacement	0.20 (0.146)	1.38	0.09 [−0.08, 0.49]	0.19 (0.152)	1.25	0.05 [−0.11, 0.49]	0.42^*^ (0.170)	2.45	0.09 [0.08, 0.75]
Interference	−0.14 (0.141)	−1.02	0.02 [−0.42, 0.13]	−0.15 (0.146)	−1.05	0.04 [−0.44, 0.13]	−0.08 (0.165)	−0.49	0.02 [−0.40, 0.24]
Complementarity	0.71^***^ (0.115)	6.17	0.02 [0.49, 0.93]	0.71^***^ (0.120)	5.93	0.21 [0.48, 0.94]	0.52^**^ (0.137)	3.83	0.14 [0.26, 0.79]

*b* = unstandardized regression coefficient; SE = standard error. *r* = Pearson’s correlation coefficient. 95% confidence intervals for bs are provided. ^***^p_bonf_ < 0.001, ^**^p_bonf_ < 0.01, ^*^p_bonf_ < 0.05.

### Does social distancing play a role In The relationship between mobile phone Use and well-being?

At the within-person level, all of the associations with phone complementarity and feeling good, calm, and energetic hold after controlling for the extent to which people socially distance (all *p*_bonfs_ < 0.0005). But does the relationship between phone use and how people feel differ depending on the extent to which they socially distance? Since the study was conducted in the early stages of the pandemic, the extent to which people varied in their social distancing from week-to-week practices was low (SD = 0.58 on our four-point scale). Accordingly, social distancing generally did not significantly interact with the phone use indicators to predict feeling good, calm, or energetic. However, social distancing did significantly interact with phone displacement to predict feeling calm. Specifically, phone displacement and social distancing significantly positively interacted to predict feeling calm (*b* = 0.63, *z* = 3.48, *p*_bonf_ = 0.002). In other words, using one’s phone to replace other activities was related to feeling calmer, especially on days when people reported social distancing more. However, given the lack of a main effect, these interactions should be interpreted with caution.

Similarly, at the between-person level, the associations between the phone use indicators and feeling good, calm, and energetic hold after controlling for social distancing (all *p*_bonfs_ < 0.05). The extent to which people varied in their social distancing practices was also low at the between-person level (SD = 0.71). Unsurprisingly, then, phone use indicators did not significantly interact with social distancing to predict feeling good, calm, or energetic.

## Discussion

We find that in a time of high social distancing during the COVID-19 pandemic, people reaped the benefits of phone use for well-being without incurring the costs associated with phone use in pre-pandemic research. Specifically, consistent with pre-pandemic research (e.g., Kushlev et al., 2017), we find that people who used their phones in a complementary way—to access information, entertainment, and connection not otherwise available—felt better, calmer, and more energetic. Furthermore, we show that the same individuals felt better, calmer and more energetic on days when they used their phones for complementary purposes. Pre-pandemic research also shows, however, that phone use often undermines well-being, especially when it displaces (Lanaj et al., 2014; Hughes and Burke, 2018) or interferes with other activities (Dwyer et al., 2018; Kushlev and Dunn, 2019). In contrast, we found no evidence that phone interference or displacement predicted lower well-being during the initial stages of the pandemic. Thus, though the pre-pandemic literature has generally linked phone use and screentime with poorer well-being (Twenge and Campbell, 2019), we find that phone use during the pandemic was associated with higher, not lower well-being.

In line with previous research, phone complementarity was related to higher levels of well-being. That is, the greater affordance to information and opportunities provided by a phone was related to people having better moods, feeling calmer, and feeling more energetic. The ease of access to information and opportunities may have become even more important during the COVID-19 Pandemic when face-to-face social contact was severely limited, which significantly increased people’s level of stress (Halliburton et al., 2021). Therefore, using one’s phone to maintain existing relationships and gain access to information may have facilitated in maintaining some semblance of pre-pandemic life, thus predicting higher well-being.

People typically feel worse when their phone use displaces activities critical for well-being, such as sleep (Lanaj et al., 2014). We find little evidence that phone displacement undermined well-being during the pandemic. This may be because there were fewer positive activities that phone use could displace during the pandemic when social activities and events were discouraged. Presumably, however, people needed just as much sleep during the pandemic as they did pre-pandemic. As lockdowns disrupted routines, sleep–wake cycles were delayed during the pandemic (Sinha et al., 2020). Thus, in the relative lack of routine during the pandemic, phone use may have been less likely to displace sleep. Finally, as the pandemic introduced new stressors, phone displacement might have been beneficial for well-being by displacing more stressful activities (Kushlev and Leitao, 2020) and introducing a welcome source of distraction (Sheppes and Meiran, 2007; Quoidbach et al., 2010).

In contrast to pre-pandemic research, we found no evidence that phone interference predicted lower well-being. Just as with displacement, this lack of effect may be due to the relative lack of rewarding activities associated with social distancing. Indeed, most previous research on the interference effects of phones has shown that phones decrease well-being precisely by interfering with face-to-face social interactions (Dwyer et al., 2018; Kushlev and Dunn, 2019). In addition, during the COVID-19 Pandemic, phones may have also interfered with activities harmful to well-being, such as rumination. Overall, then, though null findings should be interpreted with caution, our evidence suggests that phone use may not have been as harmful during the COVID-19 Pandemic.

Our findings were generally consistent with the Displacement–Interreference–Complementarity Framework: During a time of limited rewarding activities, complementary phone use continued to predict higher well-being, whereas well-documented phone interference and displacement effects were absent. According to the framework, however, at higher levels of social distancing, phone complementarity effects should have been stronger and phone displacement and interference effects should have been weaker. But we found little evidence that these effects depended on how much people socially distanced. Other research during the pandemic, however, showed that the benefits of online social interactions for well-being were greater when social distancing measures were more extreme (Marinucci et al., 2022). Specifically, online social interactions predicted lower distress only during the severe isolation stage in Italy that included prohibiting people from leaving their homes except for work and urgent health reasons. The social distancing measures that our participants in the United States experienced were much milder in comparison and participants, on average, reported high but not extreme levels of practicing social distancing (*M* = 3.12 on a scale from 1–not at all to 4–completely). Relatedly, people in our sample did not differ much in the extent to which they practiced social distancing, potentially preventing us from detecting moderating effects. Indeed, the extent to which people varied in their social distancing practices was low in this sample at both the within (SD = 0.58) and between (SD = 0.71) person levels.

This study had several important limitations that should be discussed. First, participants self-report on their levels of phone displacement, interference, and complementarity. However, people tend to misestimate the extent to which they use their phones. Future research should use more objective techniques, such as phone tracking, or peer reports in accordance with self-reports to gain a better understanding of how people are using their phones and the extent to which it relates to well-being. In addition, we used *ad hoc* measures of displacement, interference, and complementarity. Though theoretically justified, it is important for future research to develop validated measures of these constructs. For example, we measured phone displacement as the amount of time people spent on their phones in bed, the extent to which they used their phones more than they wanted to, and their total screentime. This crude measure of displacement fails to distinguish between screen time that displaces positive versus negative activities. As such, future research should utilize more precise measures of phone displacement, perhaps by explicitly asking people if they chose to use their phones over partaking in specific other activities. Furthermore, this study was conducted solely in the United States. However, other countries tend to use their phones in different ways (Langer et al., 2017) and have had different responses to the COVID-19 Pandemic (Kennedy et al., 2020). Therefore, future research should collect a more diverse sample to improve the generalizability of these results.

In sum, there is consistent evidence to suggest that using one’s phone for complementary purposes is associated with increases in well-being, as indicated by better mood, feeling calmer, and feeling more energetic, whereas spending more time on one’s phone and reporting that one’s phone interferes with daily life are generally not significantly associated with feeling good, calm, or energetic. Furthermore, we do not find consistent evidence that social distancing influences these associations. This study highlights the idea that phone use can be beneficial to individual’s well-being if it is used to complement their existing experiences.

## Data availability statement

The datasets presented in this study can be found in online repositories. The names of the repository/repositories and accession number(s) can be found at: https://osf.io/b6zdu/?view_only=1c85c73f75fb4ead99bac948c6457982.

## Ethics statement

The studies involving human participants were reviewed and approved by Institutional Review Board Georgetown University. The patients/participants provided their written informed consent to participate in this study.

## Author contributions

KK designed the studies, conceptualized the research question, and collected the data. KK and JH analyzed the data and wrote the manuscript. All authors contributed to the article and approved the submitted version.

## Conflict of interest

The authors declare that the research was conducted in the absence of any commercial or financial relationships that could be construed as a potential conflict of interest.

## Publisher’s note

All claims expressed in this article are solely those of the authors and do not necessarily represent those of their affiliated organizations, or those of the publisher, the editors and the reviewers. Any product that may be evaluated in this article, or claim that may be made by its manufacturer, is not guaranteed or endorsed by the publisher.

## Footnotes

^1^ https://doi.org/10.17605/OSF.IO/6TZGF

^2^ https://doi.org/10.17605/OSF.IO/B6ZDU

^3^In our preregistration, we stated that we plan to end the study by July 2020 when we had assumed that things would ‘return back to normal’ by then. Since the pandemic was far from over in July 2020, we continued data collection until October.

^4^ https://doi.org/10.17605/OSF.IO/6TZGF
