# Supplementary material for: Did smartphones enhance or diminish well-being during the COVID-19 pandemic?
Source: Front Psychol. 2023 Mar 13;14:1094196. doi: 10.3389/fpsyg.2023.1094196 (PMC10040682; doi:10.3389/fpsyg.2023.1094196)
Supplement: Supplementary file 1 [file Data_Sheet_1.docx]

**Supplementary Online Materials for Did Smartphones Enhance or Diminish Well-Being During the COVID-19 Pandemic?**

**Table of Contents**

Table S1a. Within-Person Correlations Between Phone Use, Social Distancing, and Well-Being…………………………………….....3

Table S1b. Between-Person Correlations Between Phone Use, Social Distancing, and Well-Being…………………..………………....3

Table S2a. Fixed Components from the Growth Models for Phone Use and Social Distancing Predicting Feeling Good Throughout the Experimental Period……………………………………………………………………………………………………………………….4

Table S2b. Random Components From the Growth Models for Phone Use and Social Distancing Predicting Feeling Good Throughout the Experimental Period..………………………………………………………………………………………………………………… 5

Table S3a. Fixed Components from the Growth Models for Phone Use and Social Distancing Predicting Feeling Calm Throughout the Experimental Period…………………………………………………………………………………………………………………….…6

Table S3b. Random Components From the Growth Models for Phone Use and Social Distancing Predicting Feeling Calm Throughout the Experimental Period..………………………………………………………………………………………………………………….7

Table S4a. Fixed Components from the Growth Models for Phone Use and Social Distancing Predicting Feeling Energetic Throughout the Experimental Period…………………………..…………………………………………………………………………………….…8

Table S4b. Random Components From the Growth Models for Phone Use and Social Distancing Predicting Feeling Energetic Throughout the Experimental Period…….……………………………………………………………………………………………..…9

Table S1a. Within-Person Correlations Between Phone Use, Social Distancing, and Well-Being

|  | Feeling Good | Feeling Calm | Feeling Energetic | Displacement | Interference | Complementarity | Social Distancing |
| --- | --- | --- | --- | --- | --- | --- | --- |
| *r* | | | | | | | |
| Feeling Good | 1.00 |  |  |  |  |  |  |
| Feeling Calm | .51*** | 1.00 |  |  |  |  |  |
| Feeling Energetic | .37*** | .44*** | 1.00 |  |  |  |  |
| Displacement | .05 | .05 | .03 | 1.00 |  |  |  |
| Interference | .00 | -.01 | .10* | .16*** | 1.00 |  |  |
| Complementarity | .24*** | .16*** | .21*** | .06 | .20*** | 1.00 |  |
| Social Distancing | .09* | .04 | .09* | .06 | .10* | .20*** | 1.00 |

*r* = Pearson’s correlation coefficient. ****p_bonf_* < .001, ***p_bonf_* <.01, **p_bonf_* <.05, †*p_bonf_* <.10

Table S1b. Between-Person Correlations Between Phone Use, Social Distancing, and Well-Being

|  | Feeling Good | Feeling Calm | Feeling Energetic | Displacement | Interference | Complementarity | Social Distancing |
| --- | --- | --- | --- | --- | --- | --- | --- |
| *r* | | | | | | | |
| Feeling Good | 1.00 |  |  |  |  |  |  |
| Feeling Calm | .63*** | 1.00 |  |  |  |  |  |
| Feeling Energetic | .61*** | .60*** | 1.00 |  |  |  |  |
| Displacement | .16† | .13 | .23** | 1.00 |  |  |  |
| Interference | .13 | .14 | .18* | .64*** | 1.00 |  |  |
| Complementarity | .39*** | .40*** | .31*** | .17† | .31*** | 1.00 |  |
| Social Distancing | .26*** | .23** | .13 | -.08 | .02 | .45*** | 1.00 |

*r* = Pearson’s correlation coefficient. ****p_bonf_* < .001, ***p_bonf_* <.01, **p_bonf_* <.05, †*p_bonf_* <.10

Table S2a. Fixed Components from the Growth Models for Phone Use and Social Distancing Predicting Feeling Good Throughout the Experimental Period

|  | Intercept-only growth model | | | Unconditional Growth Model | | | | Conditional Growth Model | | | |  |
| --- | --- | --- | --- | --- | --- | --- | --- | --- | --- | --- | --- | --- |
| Predictor | *b* | 95% CI | *p_bonf_* | | *b* | 95% CI | *p_bonf_* | | *b* | 95% CI | *p_bonf_* | |
| Intercept | 1.02*** | [0.87, 1.17] | <.001 | | 0.95** | [0.78, 1.12] | <.001 | | 1.07*** | [0.90, 1.12] | <.001 | |
| Time |  |  |  | | 0.03 | [0.00, 0.05] | 0.129 | | 0.02 | [-0.01, 0.96] | 0.597 | |
| Displacement |  |  |  | |  |  |  | |  |  |  | |
| Within |  |  |  | |  |  |  | | 0.12 | [-.009, 0.33] | 0.822 | |
| Between |  |  |  | |  |  |  | | 0.20 | [-0.10, .049] | 0.589 | |
| Interference |  |  |  | |  |  |  | |  |  |  | |
| Within |  |  |  | |  |  |  | | -0.14 | [-0.32, 0.05] | 0.455 | |
| Between |  |  |  | |  |  |  | | -0.21 | [-0.52, 0.09] | 0.525 | |
| Complementarity |  |  |  | |  |  |  | |  |  |  | |
| Within |  |  |  | |  |  |  | | 0.54*** | [0.35, 0.73] | <.001 | |
| Between |  |  |  | |  |  |  | | 0.69*** | [0.42, 0.96] | <.001 | |
| Social Distance |  |  |  | |  |  |  | |  |  |  | |
| Within |  |  |  | |  |  |  | | 0.06 | [-0.08, 0.19] | 1.238 | |
| Between |  |  |  | |  |  |  | | 0.19 | [-0.05, 0.44] | 0.397 | |
| Displacement*Social Distance |  |  |  | |  |  |  | |  |  |  | |
| Within |  |  |  | |  |  |  | | 0.09 | [-0.25, 0.44] | 1.805 | |
| Between |  |  |  | |  |  |  | | 0.30 | [-0.12, 0.71] | 0.487 | |
| Interference*Social Distance |  |  |  | |  |  |  | |  |  |  | |
| Within |  |  |  | |  |  |  | | -0.23 | [-0.51, 0.05] | 0.305 | |
| Between |  |  |  | |  |  |  | | 0.10 | [-0.31, 0.52] | 1.907 | |
| Complementarity*Social Distance |  |  |  | |  |  |  | |  |  |  | |
| Within |  |  |  | |  |  |  | | -0.12 | [-0.41, 0.17] | 1.289 | |
| Between |  |  |  | |  |  |  | | -0.36 | [-0.71, 0.01] | 0.141 | |

*Note*. *b* = unstandardized regression coefficient; 95% confidence intervals for *b*s are provided. ****p_bonf_* < .001, ***p_bonf_* <.01.

Table S2b. Random Components From the Growth Models for Phone Use and Social Distancing Predicting Feeling Good Throughout the Experimental Period

| Statistic | Intercept-only growth model | Unconditional Growth Model | Conditional Growth Model |
| --- | --- | --- | --- |
| σ^2^ | 1.24 | 1.23 | 1.14 |
| τ_00,ID_ | 0.71 | 0.73 | 0.55 |
| ICC_ID_ | 0.37 | 0.37 | 0.32 |
| *R*^2^ | .00 | .00 | .16 |
| Deviance | 2592.93 | 2595.68 | 2522.51 |
| AIC | 2598.93 | 2607.68 | 2558.51 |

Table S3a. Fixed Components from the Growth Models for Phone Use and Social Distancing Predicting Feeling Calm Throughout the Experimental Period

|  | Intercept-only growth model | | | Unconditional Growth Model | | | Conditional Growth Model | | |
| --- | --- | --- | --- | --- | --- | --- | --- | --- | --- |
| Predictor | b | 95% CI | *P_bonf_* | b | 95% CI | *P_bonf_* | b | 95% CI | *P_bonf_* |
| Intercept | 0.69*** | [0.54, 0.85] | <.001 | 0.65*** | [0.48, 0.82] | <.001 | 0.72*** | [0.54, 0.90] | <.001 |
| Time |  |  |  | 0.02 | [-0.01, 0.04] | 0.79 | 0.01 | [-0.02, 0.03] | 1.796 |
| Displacement |  |  |  |  |  |  |  |  |  |
| Within |  |  |  |  |  |  | 0.11 | [-0.10, -.33] | 0.907 |
| Between |  |  |  |  |  |  | 0.14 | [-0.16, 0.45] | 1.104 |
| Interference |  |  |  |  |  |  |  |  |  |
| Within |  |  |  |  |  |  | -0.13 | [-0.32, 0.06] | 0.545 |
| Between |  |  |  |  |  |  | -0.11 | [-0.43, 0.21] | 1.535 |
| Complementarity |  |  |  |  |  |  |  |  |  |
| Within |  |  |  |  |  |  | 0.39*** | [0.19, 0.58] | <.001 |
| Between |  |  |  |  |  |  | 0.65*** | [0.36, 0.94] | <.001 |
| Social Distance |  |  |  |  |  |  |  |  |  |
| Within |  |  |  |  |  |  | -0.03 | [-0.16, 0.45] | 2.154 |
| Between |  |  |  |  |  |  | 0.17 | [-0.09, -.43] | 0.660 |
| Displacement*Social Distance |  |  |  |  |  |  |  |  |  |
| Within |  |  |  |  |  |  | 0.63** | [0.28, 0.98] | 0.002 |
| Between |  |  |  |  |  |  | 0.42 | [-0.02, 0.85] | 0.191 |
| Interference*Social Distance |  |  |  |  |  |  |  |  |  |
| Within |  |  |  |  |  |  | -0.31† | [-0.60, -0.03] | 0.095 |
| Between |  |  |  |  |  |  | -0.22 | -0.65, 0.22] | 1.01 |
| Complementarity*Social Distance |  |  |  |  |  |  |  |  |  |
| Within |  |  |  |  |  |  | -0.11 | [-0.41, -.19] | 1.410 |
| Between |  |  |  |  |  |  | -0.13 | [-0.50, 0.24] | 1.488 |

*Note*. *b* = unstandardized regression coefficient; 95% confidence intervals for *b*s are provided. ****p_bonf_* < .001, ***p_bonf_* <.01, **p_bonf_* <.05, †*p_bonf_* <.10.

Table S3b. Random Components From the Growth Models for Phone Use and Social Distancing Predicting Feeling Calm Throughout the Experimental Period

| Statistic | Intercept-only growth model | Unconditional Growth Model | Conditional Growth Model |
| --- | --- | --- | --- |
| σ^2^ | 1.27 | 1.27 | 1.20 |
| τ_00,ID_ | 0.76 | 0.70 | 0.62 |
| ICC_ID_ | 0.38 | 0.35 | 0.34 |
| *R*^2^ | .00 | .00 | .14 |
| Deviance | 2613.98 | 2618.86 | 2568.35 |
| AIC | 2619.98 | 2630.86 | 2604.35 |

*Note*. The ID subscripts indicate between-subjects statistics. The *R*^2^ for multilevel models is the marginal variance explained, defined as the variance explained by fixed effects. AIC = Akaike information criterion.

Table S4a. Fixed Components from the Growth Models for Phone Use and Social Distancing Predicting Feeling Energetic Throughout the Experimental Period

|  | Intercept-only growth model | | | Unconditional Growth Model | | | Conditional Growth Model | | |
| --- | --- | --- | --- | --- | --- | --- | --- | --- | --- |
| Predictor | b | 95% CI | *P_bonf_* | b | 95% CI | *P_bonf_* | b | 95% CI | *P_bonf_* |
| Intercept | 0.80*** | [0.63, 0.97] | <.001 | 0.77*** | [0.58, 0.95] | <.001 | 0.90*** | [0.70, 1.09] | <.001 |
| Time |  |  |  | 0.02 | [-0.02, 0.04] | 1.143 | 0.01 | [-0.02, 0.03] | 1.664 |
| Displacement |  |  |  |  |  |  |  |  |  |
| Within |  |  |  |  |  |  | 0.04 | [-0.16, 0.24] | 2.107 |
| Between |  |  |  |  |  |  | 0.39† | [-.04, 0.73] | 0.093 |
| Interference |  |  |  |  |  |  |  |  |  |
| Within |  |  |  |  |  |  | 0.13 | [-0.05, 0.31] | -0.472 |
| Between |  |  |  |  |  |  | -0.09 | [-0.45, 0.26] | 1.842 |
| Complementarity |  |  |  |  |  |  |  |  |  |
| Within |  |  |  |  |  |  | 0.41*** | [0.23, 0.60] | <.001 |
| Between |  |  |  |  |  |  | 0.59** | [0.27, 0.91] | 0.001 |
| Social Distance |  |  |  |  |  |  |  |  |  |
| Within |  |  |  |  |  |  | 0.03 | [-0.10, 0.16] | 1.920 |
| Between |  |  |  |  |  |  | -0.04 | [-0.33, 0.25] | 2.343 |
| Displacement*Social Distance |  |  |  |  |  |  |  |  |  |
| Within |  |  |  |  |  |  | -0.02 | [-0.36, 0.31] | 2.717 |
| Between |  |  |  |  |  |  | 0.14 | [-0.33, 0.62] | 1.680 |
| Interference*Social Distance |  |  |  |  |  |  |  |  |  |
| Within |  |  |  |  |  |  | -0.25** | [-0.52, 0.02] | 0.005 |
| Between |  |  |  |  |  |  | -0.05 | [-0.53, 0.44] | 2.554 |
| Complementarity*Social Distance |  |  |  |  |  |  |  |  |  |
| Within |  |  |  |  |  |  | 0.06 | [-0.23, 0.34] | 2.071 |
| Between |  |  |  |  |  |  | -0.45 | [-0.86, -0.03] | 0.114 |

Table S4b. Random Components From the Growth Models for Phone Use and Social Distancing Predicting Feeling Energetic Throughout the Experimental Period

| Statistic | Intercept-only growth model | Unconditional Growth Model | Conditional Growth Model |
| --- | --- | --- | --- |
| σ^2^ | 1.31 | 1.10 | 1.05 |
| τ_00,ID_ | 1.07 | 1.06 | 0.97 |
| ICC_ID_ | 0.49 | 0.35 | 0.48 |
| *R*^2^ | .00 | .00 | .12 |
| Deviance | 2586.63 | 2590.45 | 2541.10 |
| AIC | 2592.63 | 2602.45 | 2577.10 |
| Family | LMM | LMM | LMM |
